# Impact of the COVID-19 pandemic on antimicrobial stewardship support for general practices in England: a qualitative interview study

**DOI:** 10.3399/BJGPO.2022.0193

**Published:** 2023-08-09

**Authors:** Anne Campbell, Aleksandra J Borek, Monsey McLeod, Sarah Tonkin-Crine, Koen B Pouwels, Laurence SJ Roope, Benedict WJ Hayhoe, Azeem Majeed, A Sarah Walker, Alison Holmes

**Affiliations:** 1 National Institute for Health Research (NIHR), Health Protection Research Unit in Healthcare Associated Infections and Antimicrobial Resistance, Imperial College London, London, UK; 2 Nuffield Department of Primary Care Health Sciences, University of Oxford, Oxford, UK; 3 Centre for Medication Safety and Service Quality, Pharmacy Department, Imperial College Healthcare NHS Trust, London, UK; 4 NIHR Imperial Patient Safety Translational Research Centre, Imperial College London, London, UK; 5 NIHR Health Protection Research Unit in Healthcare Associated Infections and Antimicrobial Resistance, University of Oxford, Oxford, UK; 6 Nuffield Department of Population Health, University of Oxford, Oxford, UK; 7 Primary Care and Public Health, Imperial College London, London, UK; 8 NIHR Oxford Biomedical Research Centre, Oxford, UK

**Keywords:** antibiotics, antimicrobial stewardship, general practice, COVID-19, qualitative research

## Abstract

**Background:**

In England, clinical commissioning group (CCG; now replaced by Integrated Care Systems [ICSs]) and primary care network (PCN) professionals support primary care prescribers to optimise antimicrobial stewardship (AMS).

**Aim:**

To explore views and experiences of CCG and PCN staff in supporting AMS, and the impact of COVID-19 on this support.

**Design & setting:**

Qualitative interview study in primary care in England.

**Method:**

Semi-structured interviews with staff from CCG and PCNs responsible for AMS were conducted at two timepoints via telephone. These were audio-recorded, transcribed, and analysed thematically.

**Results:**

Twenty-seven interviews were conducted with 14 participants (nine CCG, five PCN) in December 2020–January 2021 and February–May 2021. The study found that AMS support was (1) deprioritised in order to keep general practice operational and deliver COVID-19 vaccines; (2) disrupted as social distancing made it harder to build relationships, conduct routine AMS activities, and challenge prescribing decisions; and (3) adapted, with opportunities identified for greater use of technology and changing patient and public perceptions of viruses and self-care. It was also found that resources to support AMS were valued if they were both novel, to counter AMS ‘fatigue’, and sufficiently familiar to fit with existing and/or future AMS.

**Conclusion:**

AMS needs to be reprioritised in general practice in the post-pandemic era and within the new ICSs in England. This should include interventions and strategies that combine novel elements with already familiar strategies to refresh prescribers’ motivation and opportunities for AMS. Behaviour change interventions should be aimed at improving the culture and processes for how PCN pharmacists voice concerns about AMS to prescribers in general practice and take advantage of the changed patient and public perceptions of viruses and self-care.

## How this fits in

AMS, which is key in tackling the global health challenge of antimicrobial resistance (AMR), was expected to be adversely impacted by the COVID-19 pandemic. CCG and PCN professionals who supported general practice prescribers in AMS were provided with an intervention to facilitate practice-wide implementation of three evidence-based AMS strategies. This qualitative interview study found that AMS support to general practices was deprioritised, disrupted, and adapted during the COVID-19 pandemic, and the intervention was not used as anticipated. It is recommended that AMS be reprioritised in general practice in the post-pandemic era and within ICSs in England, and this study offers direction for doing so.

## Introduction

With over 6.6 million reported deaths until December 2022, COVID-19 (coronavirus disease 2019) has had a profound impact on global mortality.^1^ Another global health challenge of paramount significance is AMR.^2–6^ Antibiotics are used more than any other antimicrobial,^5^ and 1.27 million deaths were attributed to bacterial AMR globally in 2019.^7^ General practice antibiotic prescribing, although decreasing since 2014, typically accounts for most antibiotic prescribing in England.^8–10^ Overprescribing of antibiotics offers likely marginal, if any, patient benefit that is outweighed by potential risks of antibiotics.^11^ It is a key driver of AMR.^6,12,13^ Literature from 2018 suggested that up to 23.1% of all antibiotic prescriptions in general practice in England were overprescribed.^11^

Optimising antimicrobial use through AMS —'*promoting actions that balance both the individual’s need for appropriate treatment and the longer-term societal need for sustained access to effective therapy*'^14^— is important in tackling AMR.^6^ The National Institute for Health and Care Excellence (NICE) encourages AMS strategies in English primary care, which include the following: benchmarking individual antimicrobial prescribing against local and national rates, with regular prescriber feedback; integrating audit into local and national quality improvement programmes; education and training about AMS and AMR; decision-support systems to help decide appropriateness of alternatives to immediate antibiotics (for example, delayed prescriptions); and local and national guidance for managing common infections.^15^ AMS also includes use of communication strategies;^16^ point-of-care tests or clinical scores;^17^ and national resources, including TARGET (Treat Antibiotics Responsibly, Guidance, Education, Tools; online AMS-related resources)^18^ and Antibiotic Guardian (online pledge campaign and AMS resources).^19^

CCGs, organisations responsible for commissioning primary care services in England from 2013 to 2022,^20^ were incentivised to support primary care prescribers with AMS and help deliver sustained reductions in antibiotic prescribing since 2015.^8^ This was mostly facilitated by medicines management teams (MMTs) within CCGs, and involved pharmacists promoting AMS in general practices; for example, by providing antibiotic prescribing targets and feedback^21–23^ as part of the Quality Premium.^24^ In July 2022, with the reorganisation of healthcare services in England,^25^ CCGs were replaced by ICSs.^26^ PCNs are formal collaborations of local general practices. They were established in 2019 to encourage local practices to work collaboratively and bridge the gap between individual general practices and emergent ICSs.^27^ PCNs receive additional funding to recruit new clinical pharmacists, whose roles include ensuring prescribers conserve antibiotics according to local AMS guidance.^27,28^

Although trial evidence has shown that AMS strategies developed for general practice reduce antibiotic prescribing,^29–32^ many have not been widely and consistently used in English general practice.^33^ Review evidence has shown that no single intervention sufficiently addresses the manifold influences on antibiotic prescribing.^17,34,35^ Hence, with citizens, CCG professionals, and a range of prescribers, the ‘Antibiotic Optimisation’ intervention was co-developed.^33^ It aims to facilitate practice-wide implementation of the following three evidence-based AMS strategies: enhanced communication training; C-reactive protein (CRP) point-of-care testing (POCT); and delayed prescriptions. The following four components were promoted via the Antibiotic Optimisation website (https://antibioticoptimisation.web.ox.ac.uk/): (1) practices identify a practice-based antibiotic champion; (2) practices or champions hold at least one practice meeting on antibiotic optimisation once they receive the resources or website access; (3) champions use the implementation support website section, and clinicians use the three AMS strategy sections; and (4) clinicians use physical resources, including CRP POCT equipment and printed versions of patient leaflets and clinician handouts.^33^

The Antibiotic Optimisation resources were provided to nine high-prescribing practices in England between 2019 and 2020 and a mixed-methods evaluation was conducted.^36^ The onset of the COVID-19 pandemic occurred during this time, with the expectation that this would adversely impact antimicrobial prescribing and AMS.^8,37–39^ At the start of the pandemic, general practices in England were asked to switch to a remote-first policy, resulting in most consultations occurring by video or telephone.^40,41^ GPs perceived this move to remote consultation to increase their likelihood of prescribing antibiotics, and that CCGs had suspended their usual AMS support to practices.^39^

This study aimed to understand experiences and views of CCG and PCN prescribing advisers on how AMS support that they provided to general practices was affected by the COVID-19 pandemic, and how it can be best supported going forward.

The Antibiotic Optimisation website was also provided to CCG and PCN staff to explore their views on this resource.

## Method

This was a qualitative interview study as this methodology best explores experiences of the target group. The Standards for Reporting Qualitative Research were followed.^42^

### Setting and participants

Participants were employed by a CCG or PCN in England, in a role relevant to AMS for at least 6 months. They did not participate in the study evaluating the Antibiotic Optimisation resources.

### Sampling and recruitment

The research team aimed to recruit 5–8 high-prescribing CCGs across England. All heads or leads of MMTs within the 133 CCGs existing in November 2020 were emailed a study invitation. Sixteen responses were received, representing 22 CCGs. These were ranked according to the highest percentile in volume of antibiotic items per STAR-PU (specific therapeutic group age–sex related prescribing unit) using the most recent OpenPrescribing data^43^ available before the start of recruitment. Heads or leads of MMTs of the eight highest ranked responding CCGs were emailed and asked to circulate study documentation to relevant CCG and PCN staff.

### Data collection

One online (Microsoft Teams) 30-minute PowerPoint presentation (Supplementary Box S1) was conducted with each participating MMT in December 2020 to introduce the Antibiotic Optimisation website. Participants were advised to use the website however they wished to support general practices with AMS. They were invited to participate in up to three follow-up interviews (30, 15, and 15-minutes duration): the first planned for December 2020 (approximately 48 hours after website introduction), and then others planned for February and April 2021 (approximately 2 and 4 months later). This was to explore how participants intended to use the website and then how, if at all, they used it. Semi-structured interviews were conducted by telephone by a qualitative, non-clinical researcher (AC). The interview topic guide (Supplementary Box S2) explored participants’ views of the website and (perceived) changes in local practice prescribing, including the perceived effect of the COVID-19 pandemic on current and potential future practice; additional questions around how primary care was managing and/or assessing patients with respiratory tract infections were asked in the second interview. All participants provided consent verbally and written records of consent were made. All interviews were audio-recorded and transcribed verbatim, with transcripts checked and anonymised. Participants were offered £80 in online shopping vouchers as reimbursement for taking part.

### Data analysis

All transcripts were uploaded to NVivo (version 12) and analysed thematically.^44^ Thematic analysis was used because it allows the researcher to systematically identify patterns in interview data to answer research questions, without necessitating theoretical foundation or development. An essentialist or realist epistemological stance was taken and themes were identified on a semantic level (describing and interpreting the data on the explicit level of meaning).^44^ Three researchers (AC, AB, and MMc) independently and inductively coded four transcripts each from the first interviews, and then compared and integrated their codes. Higher-level categories and themes were identified, resulting in a hierarchical coding framework. This was used by AC to code the remaining transcripts, while adding new codes as necessary.

## Results

Responses were received from seven of the eight heads or leads of MMTs asked to circulate study documentation, which represented 13 CCGs (one team covered six CCGs, another covered two CCGs). Since the inception of ICSs, the 13 CCGs have been replaced by four ICSs. The median percentile in volume of antibiotic items per STAR-PU prescribed in August 2020 by participating CCGs was 74 (range = 35–93). Twenty-seven interviews were conducted with 14 participants (CCG = 9, PCN = 5) at two timepoints (Table 1). All 14 participants participated in the first interview; most (*n* = 12/14) occurred in December 2020 (mean duration = 31 minutes, range = 20–39) around the end of the second UK COVID-19 lockdown. Most second interviews (*n* = 9/13) occurred in February 2021 (mean duration = 19 minutes, range = 13–24) during the third lockdown.^36^ The UK COVID-19 vaccination programme began on 8 December 2020. The 7-day period with the highest number of vaccinations was 15–21 March 2021.^45^ One participant declined their second interview owing to COVID-19 pressures. The study team cancelled all third interviews to avoid adding pressure on health professionals.

**Table 1.** Participant details and interviews conducted at both timepoints

CCG or PCN	Participants per CCG or PCN	Participant ID (CCG or PCN)^a^	Interviews		Self-reported role^b^	Years in role^c^
			Timepoint 1	Timepoint 2		
A	1	A1 (CCG)	Dec 2020	Feb 2021	Head of medicines optimisation	1–3
B	3	B1 (PCN)	Dec 2020	Feb 2021	Senior PCN pharmacist	1–3
		B2 (PCN)	Dec 2020	Feb 2021	Senior PCN pharmacist	<1
		B3 (CCG)	Dec 2020	Feb 2021	Senior pharmacist	>3
C	2	C1 (CCG)	Dec 2020	Feb 2021	Prescribing adviser	>3
		C2 (CCG)	Dec 2020	Feb 2021	Practice pharmacist	<1
D	2	D1 (PCN)	Dec 2020	May 2021	Senior PCN pharmacist	<1
		D2 (CCG)	Dec 2020	Apr 2021	Pharmaceutical adviser	1–3
E	2	E1 (PCN)	Dec 2020	Feb 2021	PCN pharmacist	<1
		E2 (CCG)	Jan 2021	Apr 2021	Senior medicines optimisation pharmacist	1–3
F	2	F1 (CCG)	Dec 2020	–	Medicines optimisation technician	>3
		F2 (CCG)	Dec 2020	Feb 2021	Medicines optimisation pharmacist	>3
G	2	G1 (PCN)	Dec 2020	Feb 2021	Prescribing champion (senior GP partner)	>3
		G2 (CCG)	Jan 2021	Mar 2021	Head of medicines optimisation	>3
**Total**	**14 (9 CCG & 5 PCN**)		**14**	**13**		
			**27**			

^a^Participants from one organisation were from a commissioning support unit (CSU). CSUs provide a range of support activities to CCGs: for the purpose of this study, and for anonymity, they are treated as, and reported as, CCGs. ^b^Modified slightly to preserve anonymity. ^c^Role durations classified for representativeness and anonymity: three were >3 years (5.5, 6.5, and 20 years); one PCN was formed only 3 months before the interview, the study team decided to include this PCN despite the participant thus being in the role <6 months; this was categorised as <1 year.

CCG = clinical commissioning group. PCN = primary care network.

The findings are supported by participant quotes. The example attribution ‘D2.2-CCG’ indicates that the quoted participant is the second participant from organisation ‘D’, it is their second interview, and they are from a CCG.

### Impact of the COVID-19 pandemic on AMS support to prescribers

All participants, across both timepoints, described the deprioritisation of AMS. At timepoint 1, this was to keep general practice operational to manage staff sickness, maintain core services, and manage activities postponed during COVID-19. Most new AMS initiatives were deferred; for example, reviewing individual prescribing, CRP POCT, and educational schemes. At timepoint 2, participants described how their redeployment to operationalise COVID-19 vaccines disrupted all AMS activities:

*'COVID-19’s had a negative impact on AMR* […] *because I’ve not been able to do the work I want to do* [….] *in the last couple of months, it’s been vaccinations that’s really consumed our energies.'* (F2.2-CCG)

#### Disruptions

Participants described how one public health measure in particular, social distancing, disrupted their AMS support. This meant less opportunity to engage practice staff face to face, making it harder to build-up relationships, enforce actions, and conduct previously routine AMS activities. It also made it harder to speak out about concerns about antibiotic prescribing as there were fewer occasions for opportunistic conversations, although at timepoint 2 informal relationships and casual conversations began to resume:

*'There’s just not as many of those conversations where you can reinforce that message without it being quite a formal conversation. And that, I guess we all shy away from confrontation.'* (B1.1-PCN)

*'We have an informal catch up with the GPs after clinic* [….] *sometimes the conversation is about what’s on Netflix and people’s mental health but we do also have clinical conversations and* [….] *antibiotics would certainly come up.'* (B1.2 PCN)

Participants perceived that some antibiotic prescribing had decreased, attributing this to how social distancing had reduced community seasonal respiratory infections and consultations for acute infections. They also perceived reduced access to general practice to minimise opportunistic requests for antibiotics. Conversely, they also perceived that remote consultations, another consequence of social distancing, meant prescribers were more cautious and were overprescribing antibiotics to reduce risk of hospitalisations:

*'GPs are much more, I wouldn’t say keen, but much more as though they can’t risk a patient going into hospital* [….] *a lot more cautious prescribing and overprescribing because people are not being able to be seen.'* (B2.1-PCN)

Participants described their sympathy with the new pressured uncertain circumstances in which prescribers now worked. However, they recognised that this presented challenges to their AMS support such as how could they challenge prescribing decisions in an unprecedented emergency context with everyone trying to do their best:

*'If* [GPs] *are not seeing the patient, they’ve got to do what they’re comfortable with and, actually, they’re working in a pressured environment and under new and difficult circumstances. So, I think it’s probably harder to challenge, the decisions that people make, because, there’s no precedent for this and what we’re doing, and everyone’s trying to do their best.'* (B3.1 CCG)

#### Adaptations

Participants described how some activities continued, albeit changed, including ongoing review of data despite difficulties in using it to drive change. Audits had to fit into social distancing restrictions; for example, with feedback now done by email or telephone. Although greater use of technology and more online AMS seminars offered greater accessibility and convenience, participants perceived that learning was likely more limited. There was also more use of online meetings, but this meant more difficulty in telling who was engaged. Efforts to manage this included incentivising attendance and contribution:

*'* […] *we’ve lost that face-to-face contact, so* [….] *it’s harder to tell who’s listening or watching. Because people may go onto mute or turn cameras off. But* [….] *to incentivise, we have a record of people attending, then feedback in practice as part of that incentive to attend that meeting and contribute.'* (A1.1-CCG)

Another adaptation was to change targets (for example, of prescribing incentive scheme) to preserve previous gains, rather than driving further reductions. The rationale was that there was no point in setting unachievable targets. However, achieved targets resulted in little motivation for AMS from practices:

*'We didn't think it would be a viable achievement to practices* [driving prescribing down]*. We also didn’t want to be quite like an open season of "just do what you want" — it felt most appropriate thing was ask people just not to go backwards as opposed to go forwards.'* (A1.1-CCG)

Participants described several opportunities for strengthening their AMS support including new ways of GPs engaging patients and enabling them to access resources more easily in a pandemic context; for example, using the online platform Accurx (www.accurx.com) to share PDF leaflets with patients, and benefitting from changed patient and public perceptions of viruses and self-care during the pandemic. They envisaged promoting existing AMS strategies; for example, from TARGET (https://www.rcgp.org.uk/TARGETantibiotics), or by working with communication teams to engage patients and the public to reduce consultations. AMS support could then focus on educating GPs to use this acquired patient and public knowledge in consultations:

*'We can piggyback off into next year, this greater awareness that antibiotics are not a cure and that respiratory viruses are common and can be in self-care* [….] *patient and public engagement on that and comms around all of that and how we can build on that.'* (G2.2-CCG)

Furthermore, participants described how the perceived defensive prescribing during remote consultations was problematic for their AMS support, fearing long-term consequences of learnt liberal prescribing of junior doctors, and envisaging including re-education about this in their future AMS support:

*'I fear there’s a generation of junior doctors, who’ll be coming into practice who will maybe have a more liberal attitude to antibiotics because they’ve been taught to prescribe defensively over the last year; that’s a group we may have to re-educate a bit.'* (B3.2-CCG)

### Usefulness of the Antibiotic Optimisation resources

As AMS activities were deprioritised, the Antibiotic Optimisation resources were not used as anticipated. Participants at both timepoints therefore mostly discussed their hypothetical use, including for the expected antibiotic prescribing bounce-back once social contact and face-to-face consultations resumed. Many participants described the resources as user-friendly and useful for peer support; for example, *'spoon-feeding* [GPs who] *haven’t got time to go looking for information'* (B1.1-PCN). They perceived them as fitting in with future AMS activities, such as the forthcoming prescribing assurance framework; audits; and for national or local quality improvement initiatives (for example, Royal Pharmaceutical Society AMS pharmacist training). They anticipated directing prescribers to the resources as part of action plans to address gaps in practice identified by audit, or as a tool to help identify such gaps. PCN participants also envisaged the resources helping initiate casual conversations around AMS and how the CCG could help with this messaging:

*'I'm hoping that the prescribing leads in the CCG* [….] *can coordinate some county-wide training to get the message out and to start promoting the materials, and that we can then, as PCN practice pharmacists, reinforce those messages and use the resources* [….] *or making conversation starters to back-up those messages in day-to-day conversations around antibiotics.'* (B1.1-PCN)

Participants valued what they perceived to be novel aspects of the resources. ‘Discussing antibiotics’, particularly, was perceived as helpful to counter AMS fatigue and difficulties in responding to common prescribers' pleas of *'I’ve done all that, I know all that, and it’s made no difference*' (E2.2-CCG) and who end up '*caving-in*' (E2.2-CCG) to patients’ demands for antibiotics

*'*[Talking about antibiotics will] *be really useful and it’s something new, because you get a bit of fatigue when you’ve got the same things to say every year, which we really do about antibiotics.'* (B3.2 CCG)

Some participants considered it either not worthwhile sharing the Antibiotic Optimisation resources with experienced prescribers or envisaged discomfort in telling experienced GPs that they did not know how best to communicate to their patients:

*'… if I was to stand up in front of* [….] *a room of about 100 GPs* [….] *and say, "this is about the right words to use when discussing antibiotics", I think that wouldn't be received well.' (*C1.1-CCG)

Participants envisaged emailing their networks with the Antibiotic Optimisation weblink, incorporating it into newsletters and virtual training seminars, and using the provided slides in meetings and minutes. They also considered incorporating the resource into post-audit action plans and requiring clinicians to confirm their use of it:

*'Could say, “As part of your action plan you might wish to consider, as a practice, discussing the way you discuss antibiotics with patients, and here’s the link to a website, which can provide you with some alternative phrases,"* [….] *or could say that "all prescribers as part of action plan will view this website and sign to say if they’ve had a look at it and they're aware". '* (E1.1-PCN)

At timepoint 2, some participants described how they had shared the website. Exceptionally, one reported sharing it with 400 people through presentations to the Medicines Management Board, GP leads' meetings and by weekly newsletters sent to all types of prescribers. Several participants emphasised the need to accompany the resources with more directive information. This was to direct prescribers away from parts of the website perceived to distract from their AMS support, so they did not have to say*, ' “Yes go and look at this website” and then they’re going, “oh right, when do I get my CRP reader?” when we don’t have that available*' (A1.2-CCG). Also, this was to direct them to parts perceived to be resonant with their existing or future support, including linking Antibiotic Optimisation with already familiar resources and activities:

*'Showing* [lead prescribers] *the website won’t be much benefit for them because I don’t think they’d look at it. I was going to use the resources on it to write an email and a presentation and then have a chat with them about introducing it to their practice.* [….] *after I get buy-in from the prescribing leads* [….] *have a discussion with all the prescribers.'* (D1.1-PCN)

## Discussion

### Summary

The study found that AMS support for prescribers was deprioritised during the pandemic to keep general practices operational and to allow more time to deliver the COVID-19 vaccination programme. Furthermore, AMS was disrupted by social distancing, as having less opportunity for engaging prescribers face to face made it harder to build relationships, conduct routine AMS activities, and to speak out concerns about antibiotic prescribing. Social distancing also meant more remote consultation, which participants perceived as being associated with a lowered threshold for prescribing antibiotics. Although CCG and PCN staff were sympathetic to the new pressured circumstances in which prescribers worked, they found it particularly difficult to challenge prescribing decisions in an emergency context with everyone trying to do their best. Adaptations included greater use of technology for AMS seminars and meetings, many held by video, and changing targets to maintain reductions rather than driving further reductions. Opportunities envisaged for AMS support included ‘piggybacking’ learning from the pandemic about changed patient and public perceptions of viruses and self-care and targeting new educational activities. The study also found that resources for AMS support were valued when both novel (countering ‘fatigue’ of existing AMS messages) and familiar enough to fit with existing and/or future AMS plans, user-friendly, and useful for peer support.

### Strengths and limitations

This study occurred at a unique timepoint combining the COVID-19 pandemic, the move towards reorganisation of healthcare services in England, and the provision of new materials to help support AMS in general practice.

The main limitation was that healthcare services organisation in England changed in July 2022 and, as recruitment was from CCGs, it is unclear how AMS roles and responsibilities related to those in the new ICSs. However, similar challenges are expected to be faced by the ICSs, which will also need to reprioritise AMS. Participants’ usual AMS work was affected owing to the COVID-19 pandemic, so they did not engage with the Antibiotic Optimisation intervention as anticipated and mostly discussed hypothetical use. Considering the lack of engagement with the intervention and AMS, and COVID-19-related pressures, all third interviews were cancelled as they would have unlikely provided new information. Although the data collected at the first two timepoints were sufficient to answer the research questions, it is possible that giving participants more time to use the resources, and conducting the interviews at the third timepoint, would have provided additional insights. Also, participants self-selected to participate, so they may not be representative of all MMTs (for example, it is possible they were more engaged with AMS).

### Comparison with existing literature

Despite AMR previously receiving significant global attention,^2–4^ the finding of the present study that AMS was deprioritised during the pandemic is not unexpected.^37,38^ It aligns with perceptions of GPs on the impact on antibiotic prescribing and AMS,^39^ and of UK pharmacy antimicrobial stewardship leads.^46^ Metaphors abound in medicine, especially when conveying threat and the response to it.^47^ The ‘silent pandemic’ metaphor is increasingly used regarding AMR — in literature^5,9^ and media^48^— seemingly in the hope of driving for AMS reprioritisation the same powerful behaviour change that accompanied COVID-19. Yet, conveying AMR as ‘silent’, may tacitly encourage that silence when instead a ‘ramping-up of threat decibels’^49^ is needed. However, talking about the threat of AMR, a potentially negative or fearful message, may be insufficient, ineffective, and inappropriate unless people feel equipped to act (have ‘self-efficacy’); believe their action will be effective (‘response-efficacy’);^50^ and the risks and outcomes meaningful to people are identified and addressed in messaging to convince them to act.^51^

The findings hint at one such meaningful risk, which is the discomfort experienced by those whose job it is to support AMS in general practice when they *'shy away from confrontation*' (B1.1-PCN) in ‘challenging’ prescribers about non-optimal prescribing, especially in an unprecedented, difficult situation. Despite NICE encouraging a culture where health professionals can question colleagues’ antimicrobial prescribing as an AMS strategy,^15^ attention given to raising safety concerns in other health settings,^52,53^ and social and behavioural sciences attention to AMR and AMS,^54–57^ there has been little focus on how AMS concerns may be raised with peers in general practice. Yet, some concepts from these literatures may be transferable, and may help to explain why CCG and PCN staff shy away from confronting prescribers about AMS, at least in a pandemic context. For example, the 'voiceable concern*'*,^53^ especially regarding whether the concern is seen as *'*forgivable*'*,^41^ illustrates the ‘forgivable’ reason for missing targets; for example, prescribers were perceived as doing their best in difficult circumstances, with many changes to their work (such as moving to remote consultations) and changing guidelines that led to uncertainty and confusion, particularly early in the pandemic. The COM-B model,^58^ where behaviour is influenced by capability, opportunity, and motivation, is relevant because opportunities for casual conversations and relationship-building, usually conducive to raising concerns, were severely limited owing to social distancing, and the perceived capability or motivation for speaking out curtailed owing to dislike of confrontation.

Furthermore, the influence of 'prescribing etiquette'^57^ on how AMS concerns are raised across clinical specialties and hierarchies in hospitals may also be applicable within the reorganised primary care services in England, where 'the pharmacy voice' within the new ICSs and PCNs may be best placed to speak out about antimicrobial prescribing. The authors' previous work,^36^ has shown the importance of having adequately supported antibiotic champions with easy access to prescribers. Although PCNs receive funding for clinical pharmacists, and these are obliged to ensure prescribers conserve antibiotics according to local AMS guidance,^28^ the findings have shown that they may need to be further equipped to raise AMS queries with prescribers.

The findings also support the envisaged concern that the pandemic would necessitate adaptation of AMS.^38^ It was found that routine AMS activities, if done at all, were done differently. There was greater use of technology, also found in the survey of pharmacy antimicrobial leads,^46^ with this envisioned to strengthen AMS post-pandemic;^8^ for example, by facilitating a greater range and number of professionals to join AMS meetings and seminars. The authors also found that an AMS intervention that combines novelty, for example, new ways of discussing antibiotics, with what is familiar, has appeal in terms of overcoming the ‘fatigue’ associated with AMS. Such fatigue suggests that with too little novelty, the AMS message risks being ignored (*'I’ve done all that, I know all that, and it’s made no difference'* [E2.2-CCG]), aligning with literature around what makes something interesting.^59^

### Implications for research and practice

Going forward, there is a need to understand how to combine novel interventions and strategies with what is already familiar to help reprioritise AMS post-pandemic; for example, part of the Antibiotic Optimisation website is now incorporated into TARGET (Discussing antibiotics with patients: Overview (rcgp.org.uk)). The new organisational structure of health care in England presents opportunities for behaviour change interventions to be aimed at improving the culture and processes for how PCN pharmacists voice concerns about AMS to prescribers in general practice. Innovative technology can potentially improve multidisciplinary working around AMS. The new knowledge gained through the pandemic by patients and public that antibiotics do not work for viruses and of self-care could be used to refresh existing communication strategies.

Further research should focus on how stakeholders in primary care perceive the threat of AMR and its potential AMS solutions; who is best placed to speak out about this threat and solutions; what fears or facilitators there are to speaking out; and how those speaking out may be best equipped.

In conclusion, AMS needs to again be a priority for behaviour change in the fight against AMR and be resilient to change in organisational structure (for example, the reorganisation of English health care) and to new public health challenges (for example, the COVID-19 pandemic or any other infectious disease outbreak).

## Data

The data presented in this study are available on request from the corresponding author.
